# Letter from Dr. C. Johnston

**Published:** 1857-01

**Authors:** Chr. Johnston

**Affiliations:** Lecturer on Microscopical and Comparative Anatomy of the Teeth, in Baltimore College of Dental Surgery.


### ARTICLE IV.

### *Letter from* Dr. C. Johnston.

Baltimore, *Dec.* 2, 1856.

To the Editors of the American Journal of Dental Science.

Gentlemen:

It gives me much pleasure to furnish you with the description you ask of a Microscopical preparation I recently exhibited to the College Class.

Desiring to make a fine section of *enamel,* transversely to the axis of a tooth, I selected a first left upper molar, which had been extracted from the mouth of a young lady, to make place for an artificial set; although at first, I hesitated to undertake the laborious task, inasmuch as the tooth bore, near the middle of the grinding surface, a gold filling of several years standing. I feared lest the dental structures might be (for my purpose) injured by the operative manipulation and by intrinsic change around the gold; but I was curious to ascertain whether the filling, inserted by Prof. Maynard, of Washington, and which presented a faultless appearance, would retain its position in the tooth-section, when the latter had attained its utmost thinness.

After some hours of patient work, I produced the specimen in question. It is cut, as I have remarked, transversely to the axis of the tooth, through the enamel, and the very extremities of the *dentine cusps.* It is about the $\frac{1}{550}$th of an inch in thickness, perfectly flat, smooth, and polished on both sides, and is mounted in Canada balsam. Notwithstanding the rough usage to which the section was subjected upon a corundum wheel, various hones, and a kid buffer, the filling was not even started from its position.

Examined under a magnifying power of 280 diameters, (Spencer's,) the enamel structure is beautifully conspicuous; and what is very interesting, as well as remarkable, *no interval* can be detected between the even margin of the former cavity and the solid uniform gold.

I beg leave to add that it is unnecessary and extremely difficult to reduce a *transverse section* of enamel to a lesser thickness than that given above, because the columns hold together by so feeble a tenure that the piece is apt to crumble: but I am in the habit of making *longitudinal sections* of dentine and enamel which do not exceed the $\frac{1}{1000}$th of an inch; and of dentine and cement, in any direction, not surpassing the $\frac{1}{1500}$th of an inch.

You also request me to mention the microscopic *ambrotypes* and photographs (on paper) that I use as *illustrations* in the Lectures I have the honor of giving before the dental class.

These faithful pictures were made by my friend, George B. Coale, Esq. and myself, by the assistance of Spencer's 1 inch, $\frac{1}{2}$ inch, and $\frac{1}{4}$ inch objectives, and from my own preparations. The accompanying specimens will exhibit to you in beautiful detail the exquisite structures of various teeth, of vegetable ivory, of bone and of insects, and the appearance presented by crystals. You will not fail to remark the ambrotypes of *infusoria*, rendered with all their markings, as Coconeis, Coscinodiscus, Arachnoidiscus Japonicus, and particularly the finely dotted Pleurosigma Formosum. The scale of Lepisma, although very distinctly marked, is far less difficult than the last named "Navicula."

I am, very respectfully, yours, &c.

CHR. JOHNSTON, M. D.

**Lecturer on Microscopical and Comparative Anatomy of the Teeth, in Baltimore College of Dental Surgery.**

